# Carbon-Silica Composite as Adsorbent for Removal of Hazardous C.I. Basic Yellow 2 and C.I. Basic Blue 3 Dyes

**DOI:** 10.3390/ma14123245

**Published:** 2021-06-11

**Authors:** Małgorzata Wiśniewska, Monika Wawrzkiewicz, Magda Onyszko, Magdalena Medykowska, Agnieszka Nosal-Wiercińska, Viktor Bogatyrov

**Affiliations:** 1 Department of Radiochemistry and Environmental Chemistry, Faculty of Chemistry, Institute of Chemical Sciences, Maria Curie- Sklodowska University in Lublin, M. Curie-Sklodowska Sq. 3, 20-031 Lublin, Poland; m.medykowska@poczta.umcs.lublin.pl; 2 Department of Inorganic Chemistry, Faculty of Chemistry, Institute of Chemical Sciences, Maria Curie-Sklodowska University in Lublin, M. Curie-Sklodowska Sq. 2, 20-031 Lublin, Poland; m.wawrzkiewicz@poczta.umcs.lublin.pl (M.W.); m.tarczyluk97@gmail.com (M.O.); 3 Department of Analytical Chemistry, Faculty of Chemistry, Institute of Chemical Sciences, Maria Curie-Sklodowska University in Lublin, M. Curie-Sklodowska Sq. 3, 20-031 Lublin, Poland; anosal@poczta.umcs.lublin.pl; 4 Chuiko Institute of Surface Chemistry, National Academy of Sciences of Ukraine, General Naumov Street 17, 03164 Kyiv, Ukraine; vbogat@ukr.net

**Keywords:** dyes removal, basic blue 3, basic yellow 2, adsorption, composite, wastewater treatment

## Abstract

Treatment of wastewaters containing hazardous substances such as dyes from the textile, paper, plastic and food industries is of great importance. Efficient technique for the removal of highly toxic organic dyes is adsorption. In this paper, adsorptive properties of the carbon-silica composite (C/SiO_2_) were evaluated for the cationic dyes C.I. Basic Blue 3 (BB3) and C.I. Basic Yellow 2 (BY2). The sorption capacities were determined as a function of temperature (924.6–1295.9 mg/g for BB3 and 716.3-733.2 mg/g for BY2 at 20–60 °C) using the batch method, and the Langmuir, Freundlich and Temkin isotherm models were applied for the equilibrium data evaluation using linear and non-linear regression. The rate of dye adsorption from the 100 mg/L solution was very fast, after 5 min. of phase contact time 98% of BB3 and 86% of BY2 was removed by C/SiO_2_. Presence of the anionic (SDS), cationic (CTAB) and non-ionic (Triton X-100) surfactants in the amount of 0.25 g/L caused decrease in BB3 and BY2 uptake. The electrokinetic studies, including determination of the solid surface charge density and zeta potential of the composite suspensions in single and mixed adsorbate systems, were also performed. It was shown that presence of adsorption layers changes the structure of the electrical double layer formed on the solid surface, based on the evidence of changes in ionic composition of both surface layer and the slipping plane area. The greatest differences between suspension with and without adsorbates was obtained in the mixed dye + SDS systems; the main reason for this is the formation of dye-surfactant complexes in the solution and their adsorption at the interface.

## 1. Introduction

Industrial wastewater is one of the most dangerous sources of environmental pollution. The rapid expansion of industrial production, which uses large amounts of water, has contributed significantly to pollution and, in many cases, contamination of surface waters [1]. The group of industrial wastewaters that poses serious problems in its disposal includes wastewater-containing dyes, which are difficult to decompose and poorly biodegradable. In addition, their presence significantly deteriorates the color of the wastewater, and even small concentrations in the environment cause change in the color of water reservoirs. Strong coloration of water significantly reduces light transmission, and thus the process of the photosynthesis of aquatic plants, and limits self-purification of water. Moreover, most dyes have a negative effect on aquatic animals, mainly fish and plankton. Indirect impact is related to the deterioration of living conditions by changing the light exposure and water composition. The direct effect, on the other hand, is based on the poisonous properties of some dyes [2]. Difficulties in developing an economical, effective and simple method of dye removal are caused by rapid changes in the production technology and the use of various dyes in the technological process. The main sources of dyes in industrial wastewater are the following plants: organic dyes production, textile, leather and paper industries, plastics, food, petroleum, printing and cosmetics industries.

One of the available methods of water purification from heavy metal ions is adsorption [3]. Due to the highly developed surface area (even a few thousands of m^2^/g) and porous structure, as well as favorable surface properties, carbon materials (carbon nanotubes, graphene oxide and activated carbon) are perfectly suited as effective and selective adsorbents. Their adsorptive properties can be additionally modified by formation of composites with other compounds. These combined materials composed of two or more constituents have specific characteristics and better properties than individual components.

Adsorbents containing carbon such as carbon nanotubes, activated carbon, biochar, graphene, carbon nanofibers and its derivatives are widely used in the process of removal of undesirable, toxic and hazardous substances (such as heavy metal ions, dyes, pesticides, drugs, polymers, surfactants) from aqueous solutions [4,5,6,7,8,9,10,11]. The kind of adsorption depends on the type of surface functional groups on the carbon materials and their interactions with adsorbate molecules. The main mechanisms include: ion exchange, chemical bonding, electrostatic interaction, surface complexation, surface precipitation and physical adsorption [12].

The interesting type of adsorbents are the composites of carbon with inorganic oxides (such as SiO_2_, Al_2_O_3_ and TiO_2_) or metallic elements (such as Si/C/Mn/Fe and Si/C/Mn). Previously performed studies confirmed the usefulness of the hybrid carbon-mineral nanocomposites with metallic elements (especially Si/C/Mn) for the adsorption removal of Cu(II) ions from aqueous solution in poly(acrylic acid) presence [13]. In turn, the carbon-silica composite showed high efficiency of Cu(II) adsorption from the solution containing proteins with different internal stability (ovalbumin and lysozyme) [14], as well as ionic polyacrylamides [15].

In the present study, the sorption properties of carbon-silica composite for the cationic dyes, i.e., C.I. Basic Yellow 2 and C.I. Basic Blue 3 from liquid phase, were examined. The dyes are used mainly in the textile industry, so large amounts are present in textile effluents. C.I. Basic Yellow 2 is used for silk, acrylic, tannin mordant dyeing, and also can be used for leather, paper, hemp and glue coloring. It can also be found in oil and fat, paint color, and ink. C.I. Basic Blue 3 is applied for wool/nitriles and stick/acrylic blended fabric dyeing, as well as for direct printing of acrylic carpets. That is why their effective removal is highly necessary due to the intense color of these substances and effluents. Basic dyes show significant toxicity to living organisms, especially mutagenic and carcinogenic effects [16,17]. The mechanisms of these dyes removal were characterized based on analysis of the sorption capacities, solid surface charge density and zeta potential in the presence of surfactant with different ionic character (cationic CTAB, anionic SDS and nonionic Triton X-100). The conducted research undoubtedly enriched the knowledge about the possibility of using an innovative carbon-silica composite for dye removal. So far, its suitability for separation of heavy metal ions and polymers (both natural and synthetic) has been examined [14,15]. This paper describes for the first time the use of C/SiO_2_ composite for adsorptive removal of basic dyes.

## 2. Materials and Methods

### 2.1. Composite and Dyes

**Figure 1:**
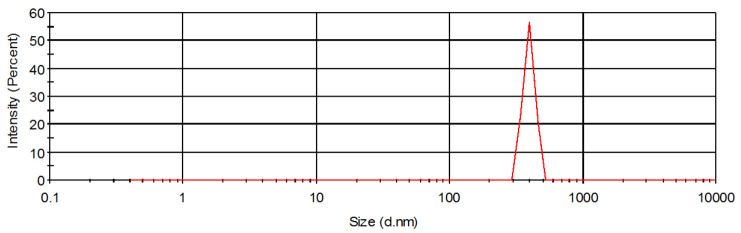
Particle size distribution of C/SiO_2_ composite.

Pyrogenic silica A-300 (Pilot plant of Institute of Surface Chemistry NAN of Ukraine, Kalush) and phenol-formaldehyde resin of novolac type (JSC ‘Ukrainian resins’) were used for composite synthesis. Both components were mixed in a weight ratio of 1:1 and ground by a porcelain ball mill for 2 h. Next these reagents were subjected to pyrolysis in argon flow at 800 °C for 2 h. The detailed textural and surface characteristics of obtained C/SiO_2_ composite was presented in previous paper [14]. The obtained product contains 31.5% (by weight) of carbon, has a specific surface area (*S_BET_*) equal to 297 m^2^/g and mean pore diameter—6.1 nm. The particle size distribution of C/SiO_2_ composite was measured using Zetameter Nano ZS apparatus (Malvern Instruments, Malvern, UK) and is presented in Figure 1. The mean diameter of solid aggregates in 0.001 mol/L NaCl supporting electrolyte solution was about 790 nm.

**Figure 2:**
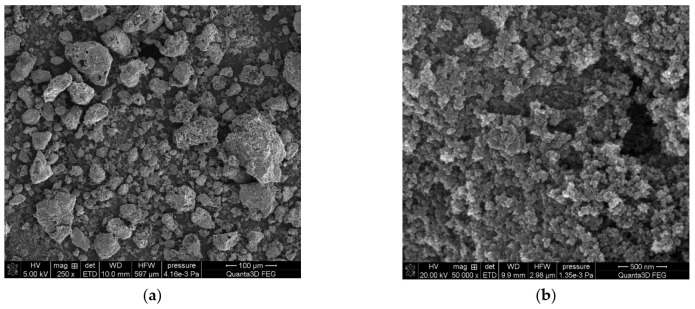

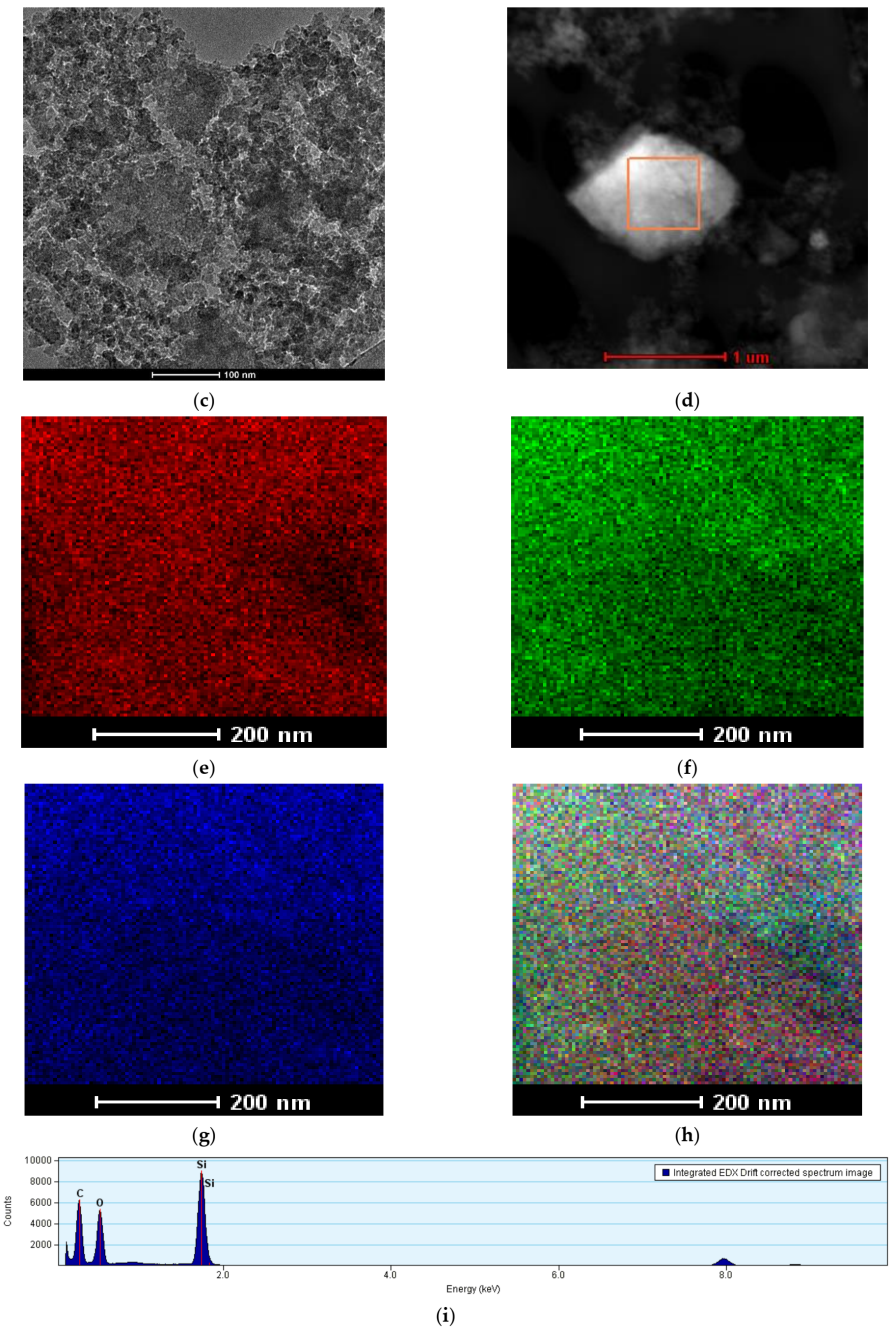
(**a**–**b**) SEM and (**c**–**d**) TEM images of C/SiO_2_ composite as well as TEM maps of (**e**) carbon, (**f**) silicon, (**g**) oxygen, (**h**) composite and (**i**) integrated EDX drift corrected spectrum.

The morphology of the C/SiO_2_ composite surface was observed using electron microscopes: scanning electron microscope (SEM) QuantaTM 3D FEG (FEI Company, Hillsboro, OR, USA) and transmission electron microscope (TEM) TecnaiTM G2 20 (FEI Company, Hillsboro, OR, USA). The appropriate detectors (EDS, EDX) cooperating with the microscopes enables the determination of element composition in the adsorbent phase. The images of C/SiO_2_ composite surface are presented in Figure 2. Analysis of the microscopic images reveals that the composite has a disordered structure in which the individual components are randomly distributed.

**Figure 3:**
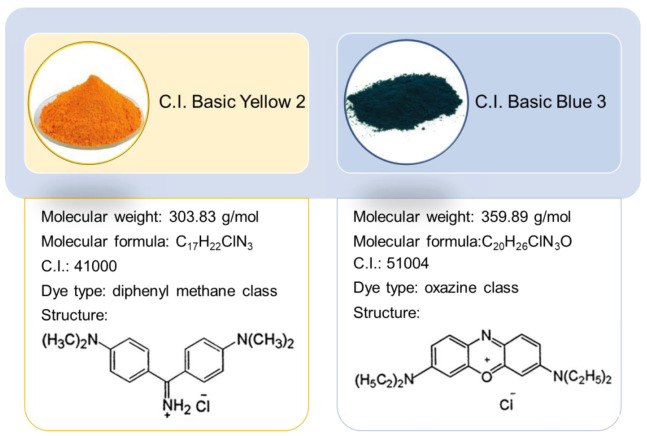
Dyes characteristics.

C.I. Basic Yellow 2 and C.I. Basic Blue 3 properties are presented in Figure 3. The dyes were purchased from Classic Dyestuff Inc. (High Point, NC, USA). The stock solutions of BY2 and BB3 of the initial concentration C_0_ = 1000 mg/L were prepared. The working solutions were prepared by diluting the stock solution in the presence of 0.001 mol/L NaCl as supporting electrolyte.

Surface active agents such as anionic sodium dodecyl sulphate (SDS), non-ionic 2-[4-(2,4,4-trimethylpentan-2-yl)phenoxy]ethanol (Triton X-100) and cationic hexadecyltrimethylammonium bromide (CTAB) of laboratory grade were purchased from Sigma-Aldrich (Darmstadt, Germany). Sodium chloride and hydroxide were obtained from Avantor Performance Materials Poland S.A. (Gliwice, Poland).

### 2.2. Adsorption Studies

In the batch adsorption experiment, 0.02 g of C/SiO_2_ was added to 20 mL of an aqueous dye solution and shaken using an Elpin 358+ (Elpin, Lubawa, Poland) laboratory shaker at 180 cycle/min. for 24 h (equilibrium experiment) at 20, 40 and 60 °C or from 1 to 240 min. (kinetic experiment) at 20 °C. The initial BY2 or BB3 concentrations used to collect isotherm data were ranged from 100 to 1000 mg/L. In order to calculate kinetic parameters in C/SiO_2_-BY2 and C/SiO_2_-BB3 systems, a dye solution with an initial concentration equal to 100 mg/L was prepared. Impact of surfactants such as SDS, CTAB and TX100 on the dye’s uptake by C/SiO_2_ after sorption time t = 60 min was investigated in the systems containing 100 mg/L of the dye and 0.25 g/L of SDS, TX100 or CTAB. The amounts of BY2 or BB3 adsorbed by C/SiO_2_ at equilibrium (q_e_) and at time t (q_t_) were calculated from the following formulas:

(1) $$q_{e}=\frac{\left(C_{0}-C_{e}\right)}{m}\times V$$

(2) $$q_{t}=\frac{\left(C_{0}-C_{t}\right)}{m}\times V$$

where: C_0_, C_e_ and C_t_ (mg/L)—dyes concentrations in the solution before adsorption, at equilibrium and after sorption time t, respectively, V (L)—volume of the solution, and m (g)—mass of C/SiO_2_.

Dye solutions were filtered after sorption experiments and dyes concentrations were measured using the UV-Vis spectrophotometry Cary 60 (Agilent, Santa Clara, CA, USA) at the maximum wavelengths 430 nm and 645 nm for BY2 and BB3, respectively. All adsorption experiments were performed in triplicate, and the mean values have been used for data evaluation. The standard deviation did not exceed 3%.

#### 2.2.1. Isotherm Studies

The equilibrium adsorption data of BY2 and BB3 on C/SiO_2_ composite were analyzed by calculation of isotherm parameters. The Langmuir, Freundlich and Temkin isotherm equations were taken into account using their linear and non-linear forms which are presented in Table 1 [18,19,20]. The Langmuir isotherm model suggests that adsorbate uptake occurs on a homogeneous surface of adsorbent, forming a monolayer without interaction between adsorbed molecules. The Freundlich and Temkin isotherm models consider surface heterogeneity and the interactions between adsorbents and adsorbates. One of the assumptions of Temkin’s model is that the heat of adsorption of all molecules in the layer decreases linearly.

Applying linear regression, the isotherm parameters were calculated from the slopes and intercepts of the C_e_/q_e_ vs. C_e_, log q_e_ vs. log C_e_ and q_e_ vs. ln C_e_ plots (Q_o_ = 1/slope, b = slope/intercept, 1/*n* = slope, k_F_ = 10^intercept^, b_T_ = RT/slope, A = exp(intercept/slope)). Microsoft Excel 2013 with the Solver add-in was used for calculation of the above mentioned parameters by non-linear regression. Calculation of the values of determination coefficients (R^2^), adjusted R-squared (adj-R^2^) and Marquardt’s percent standard deviation (MPSD) enable an assessment of the best fit of the experimental data. Adj-R^2^ is usually used to compare model of different sizes; in contrast to R^2^, it can go up or down when a variable is taken out of the model. These parameters were calculated from the following Equations (9)–(11) [19,20]:

(9) $$R^{2}=1-\frac{\sum {\left(q_{e\;exp}-q_{e\;cal}\right)}^{2}}{\sum {\left(q_{e\;exp}-q_{e\;mean}\right)}^{2}}$$

(10) $$R_{adj}^{2}=1-\left[\frac{\left(1-R^{2}\right)\left(n-1\right)}{n-k-1}\right]$$

(11) $$MPSD=\overset{n}{\underset{i=1}{\sum }}{\left(\frac{q_{e\;exp}-q_{e\;cal}}{q_{e\;exp}}\right)}_{i}^{2}$$

where: q_e exp_ (mg/g)—amount of acid dyes sorbed at equilibrium, q_e cal_ (mg/g)—amount of acid dyes sorbed calculated from the non-linear models, q_e mean_ (mg/g)—measured by the mean of q_e exp_ values, n—number of points in data sample, k—number of independent regressors.

#### 2.2.2. Thermodynamic Studies

The thermodynamic parameters of BB3 and BY2 adsorption on C/SiO_2_ were calculated at three different temperatures using the Equations:

(12) $${∆G}^{𝑜}=-RTlnK_{c}c^{0}$$

(13) $$K_{c}=\frac{q_{e}}{C_{e}}$$

(14) $$lnK_{c}c^{0}=\frac{∆S^{𝑜}}{R}-\frac{∆H^{𝑜}}{RT}$$

where Δ*G^𝑜^*—change of free energy of the system (kJ/mol), Δ*H^𝑜^*—change of free enthalpy of the system (kJ/mol), Δ*S^𝑜^*—change of free entropy of the system (kJ/mol K), *R*—gaseous constant (8.314 J/mol K), *T*—temperature (K), *K_c_*—distribution constant at equilibrium (L/g), *c^0^ ≡* 1000 g/L [21]*, q_e_*—the amount of basic dye adsorbed per unit mass of C/SiO_2_ at equilibrium (mg/g), *C*_e_—the equilibrium concentration of solution (mg/L).

### 2.3. Electrokinetic Studies

The electrokinetic measurements included determination of the solid surface charge density (*σ*_0_) and zeta potential (*ζ*) of the systems without and containing dyes or dye + surfactant mixture in the supporting electrolyte solution (0.001 mol/L NaCl). The following suspensions were examined: C/SiO_2_ + NaCl, C/SiO_2_ + NaCl + D, C/SiO_2_ + NaCl + D + CTAB, C/SiO_2_ + NaCl + D + SDS and C/SiO_2_ + NaCl + D + TX-100 (where D: BY2 or BB3). The concentration of the dyes were 100 mg/L, whereas that of the surfactants were 0.25 g/L.

A potentiometric titration method was applied to the determination of surface charge density of the examined systems and their points of zero charge (pzc). The pzc is at such pH value at which the concentration of positively and negatively charged surface groups is the same and thus *σ*_0_ = 0. Dependencies of *σ*_0_ as a function of solution pH (changing in the range 3–11) were calculated by the special software “titr_v3” [22]. The examined solution (of volume 50 mL) was introduced to the Teflon vessel thermostated (20 °C) using thermostat RE 204 (Lauda, Germany). The glass and calomel electrodes (Beckman Instruments) as well as pH-meter PHM 240 (Radiometer, Sweden) were used to continuous control of system’s pH during titration, which was performed by the use of automatic microburette Dosimat 765 (Metrohm, Switzerland) and computer. The examined systems were titrated with NaOH solution of concentration 0.1 mol/L and the applied solid mass was 0.1 g.

Electrophoretic mobility (*u_e_*) measurements allowed the determination of zeta potential (ζ) of carbon-based silica particles (Zetameter Nano ZS, Malvern Instruments, Malvern, UK) based on the Henry’s equation [23]. Moreover, the determination of isoelectric points (iep) of the solid is possible. It is at a pH value at which the concentration of positively and negatively charged groups/ions in the slipping plane area is the same and thus ζ = 0. The suspensions were prepared by addition of 0.02 g of the C/SiO_2_ composite to 200 mL of the supporting electrolyte solution without or containing one or mixed adsorbates. This prepared system was sonicated for 3 min and divided into several parts, differing in the pH value (changing in the range 2–10). Next the electrophoretic mobility of each sample was measured using dip cell in the cycles of five repetitions. Electrokinetic measurements were performed in fivefold and the mean values have been used for data estimation. The standard deviation did not exceed 3%.

## 3. Results

### 3.1. Isotherm Studies

**Figure 4:**
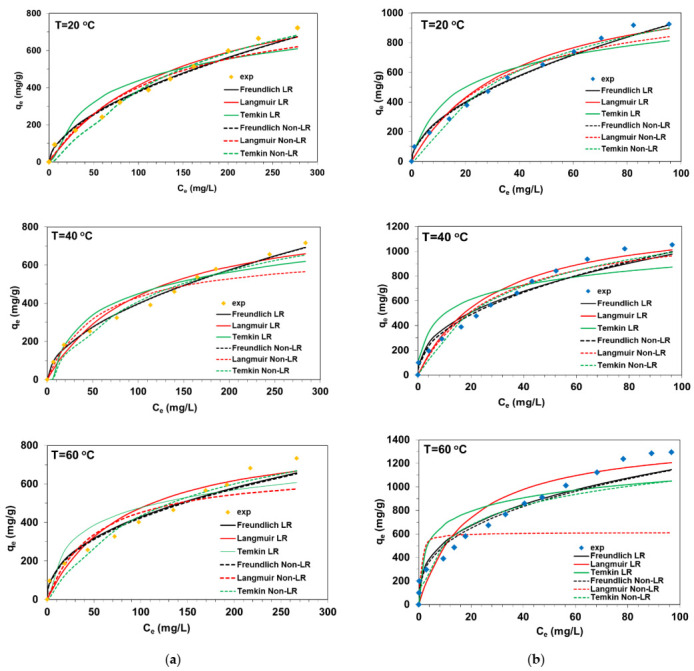
Equilibrium adsorption data in (**a**) BY2-C/SiO_2_ and **(b)** BB3-C/SiO_2_ systems at different temperatures (where: LR—linear regression, Non-LR—non-linear regression).

The equilibrium adsorption data describing BY2 and BB3 adsorption on C/SiO_2_ presented in Figure 4 were analyzed by using the linear and non-linear forms of three popular isotherm models. The isotherm parameters were calculated and are listed in Table 2 and Table 3.

Analyzing equilibrium data presented in Figure 2, the preferential adsorption of BB3 by C/SiO_2_ can be seen. The sorption capacities of C/SiO_2_ determined experimentally (q_e exp_) for BB3 were found to be 924.6 mg/g, 1052.1 mg/g and 1295.9 mg/g at 20, 40 and 60 °C, respectively. Insignificant impact of temperature on the BY2 uptake was observed, the values of q_e exp_ were ranged from 716.3 mg/g to 733.2 mg/g at 20–60 °C. The sorption capacities of the composite for BB3 and BY2 were compared with data available in the literature [17,24,25,26,27,28,29,30,31] and are presented in Table 4. In addition, many valuable reports can be found in the literature on the adsorptive removal of dyes of different types using composites [32,33,34,35,36]. It is worth mentioning here that attempts to remove BB2 and BB3 from model solutions and wastewater were made using the following methods: electrocoagulation [37], electrolysis [38], photocatalysis [39], oxidation [40], or sonification [41].

Comparing the Freundlich isotherm parameter k_F_ determined in BB3-C/SiO_2_ and BY2-C/SiO_2_ systems with the linear (29.32–243.99 mg^1−1/*n*^ L^1/*n*^/g) and non-linear (27.37–223.25 mg^1−1/*n*^ L^1/*n*^/g) regression, it was found that these values are close to each other. Table 2 and Table 3 show that 1/n heterogeneity factors are below 1 which means a favorable adsorption of a physical nature during the basic dye adsorption on the composite (e.g., hydrogen bonds between N atoms of the dyes and –OH or –OH_2_^+^ groups present on composite surface) [42,43]. The Marquardt’s percent standard deviation values varied from 0.032 to 0.365 and were the smallest precisely for the Freundlich model. Even though the Freundlich model gives better comparative numerical results (in terms of deviations), visual analysis of the data reveals that it is still only an approximation of the actual process characteristics. The values of the Langmuir isotherm parameters corresponding to the monolayer coverage of adsorbate on the adsorbent surface (Q_0_) and free energy of adsorption (k_L_) determined by the linear and non-linear method were significantly different from each other. Based on the comparison of the R^2^, adj-R^2^ and MPSD values, it can be stated that the Langmuir model does not adequately describe the adsorption systems under discussion. The Temkin model also cannot be used to describe the adsorption systems. The non-linear regression is more appropriate for the evaluation of isotherm parameters than the linear regression in the BB3-C/SiO_2_ and BY2-C/SiO_2_ systems.

### 3.2. Thermodynamic Parameters

**Figure 5:**
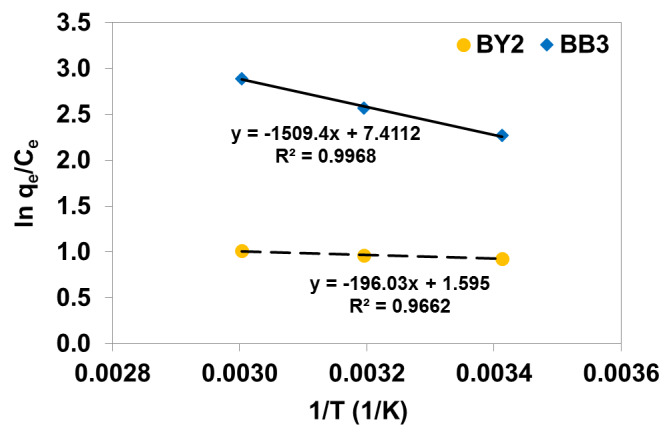
The van’t Hoff plot for calculation of enthalpy and entropy in BB3–C/SiO_2_ and BY2–C/SiO_2_ systems.

To study the feasibility of BB3 and BY2 adsorption on the composite, the thermodynamic parameters such as free energy, enthalpy and entropy changes were calculated from Equations (12) and (13). The entropy and enthalpy were evaluated from the van’t Hoff plot presented in Figure 5 and are listed in Table 5.

The negative values of ∆G^𝑜^ at 20, 40 and 60 °C show that the sorption process of basic dyes on C/SiO_2_ was spontaneous. It was reported previously by Muhammad et al. The authors of [17] studied BY2 removal by PANI/Fe_3_O_4_ composite that the ∆G^𝑜^ values in the range of −20 to 0 kJ/mol show physiosorption. The ∆G^𝑜^ values were ranged from −27.1 to −22.4 kJ/mol for BB3—C/SiO_2_ systems and from −21.9 to −19.2 kJ/mol for BY2 adsorption on C/SiO_2_, which suggest spontaneous and physical adsorption. The positive values of ∆H◦ confirmed endothermic nature of the process. The values of ∆H◦ below 84 kJ/mol also indicates physical nature of interactions between adsorbates and adsorbent [17]. The adsorption enthalpy change for BB3 is larger than that of BY2. It means that the interactions between BB3 and C/SiO_2_ surface is stronger and leads to an enhanced adsorption. Moreover, the positive ∆S^𝑜^ values 61.6 kJ/mol and 13.3 kJ/mol for BB3 and BY2, respectively) implied irregular increase in randomness at C/SiO_2_—basic dyes interface during adsorption.

### 3.3. Phase Contact Time and Surfactant Impact on Basic Dye Adsorption

**Figure 6:**
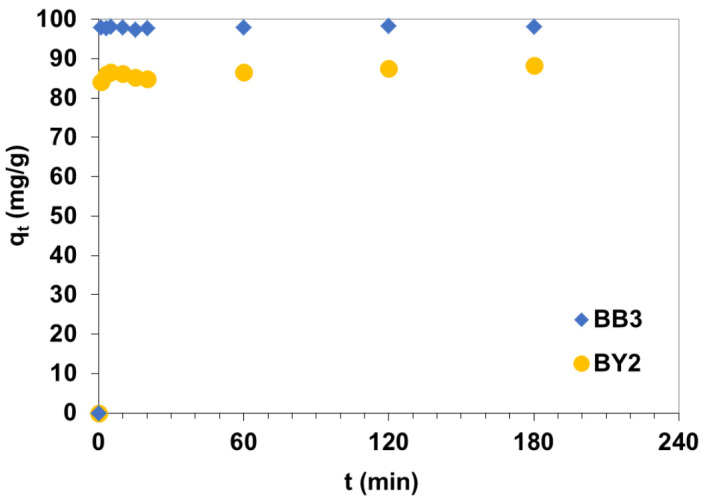
Influence of phase contact time on BB3 and BY2 uptake by C/SiO_2_ from aqueous solutions of the initial concentration 100 mg/L.

The uptake of BB3 and BY2 from aqueous solutions of the initial concentration of dye 100 mg/L as a function of contact time is shown in Figure 6.

The dye removal rate was very rapid initially and then equilibrium was reached and no significant increase in the rate of removal was observed. During 5 min of phase contact time more than 98% of BB3 (q_t_ = 98.1 mg/g) and 86% (q_t_ = 86.59 mg/g) of BY2 was uptaken by C/SiO_2_. The amounts of BB3 and BY2 adsorbed at equilibrium were found to be 98.5 mg/g for BB3 and 88.3 mg/g for BY2. The time necessary to reach the saturation was 60 min for the systems under discussion. The equilibrium time of 50 mg/L BB3 adsorption was reached within 50 to 60 min for Fe_3_O_4_, PANI, and PANI/Fe_3_O_4_ composite [17]. The amount of BB3 retained by the sulfuric acid activated montmorillonite mineral increased reached the maximum value (85–99 mg/g for different initial pH values) after approximately 60 min. Goscianska et al. The author of [31] reported that the time necessary to reach dynamic equilibrium during 75 mg/L BY2 adsorption onto the mesoporous carbons of regular structure subjected to oxidation by ammonium persulfate at 30, 60 or 100 °C was 100 min.

**Figure 7:**
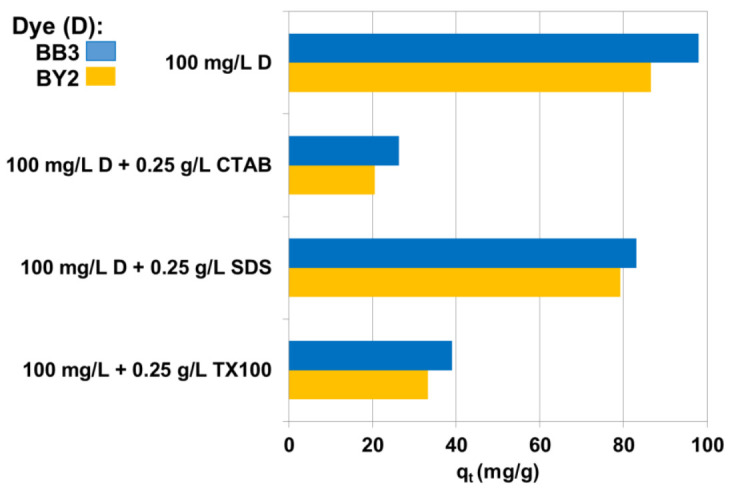
Impact of anionic (SDS), cationic (CTAB) and non-ionic (TX100) surfactants on dyes uptake by C/SiO_2_.

In the textile industry, surfactants are used as antistatic, untangling and softening agents in different processes of textile manufacturing such as scouring, dyeing and finishing. In the aforementioned textile treatment processes, the surfactant does not bind permanently to the fiber. They enter the wastewaters in the quantities in which they were introduced into the dyeing bath. They are present in wastewater together with dyes and affect the efficiency of their removal, often being a threat to aquatic ecosystems themselves. It was stated that all anionic surfactants are classified harmful for LC_50_ between 10–100 mg/L while non-ionic surfactants are toxic for LC_50_ between 1.0 and 10 mg/L [42,43,44]. Therefore, the important aspect of the paper was to investigate the dyes removal by the composite from the solutions containing 0.25 g/L of surfactants. Figure 7 compare 100 mg/L BB3 or BY2 uptake by C/SiO_2_ in the presence of anionic (SDS), cationic (CTAB) and non-ionic (TX100) surfactants.

There were observed decrease in BB3 and BY2 adsorption in presence of the surfactants. The most noticeable change was observed in the system containing CTAB. The drop of q_t_ values in this case can be explained by competitive sorption of cationic surfactant in comparison with cationic dyes. Anionic molecules of SDS interact with cationic dyes. Therefore, the concentration of free dye cations in solution decreases and BB3 and BY2 adsorption decreases. However, this does not preclude the adsorption of dyes-SDS complexes on C/SiO_2_ which can be found by electrokinetic studies.

### 3.4. Electrokinetic Results

**Figure 8:**
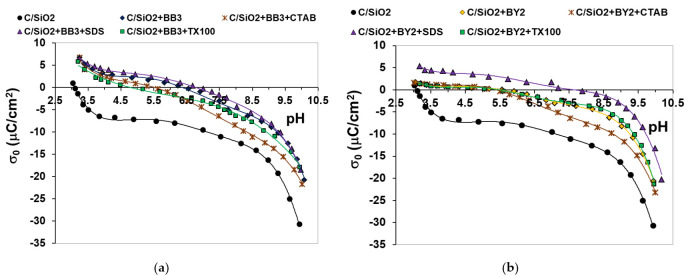
(**a**) Dependencies of the C/SiO_2_ surface charge density vs. solution pH in the systems containing BB3 dye and surfactants with different ionic character; (**b**) Dependencies of the C/SiO_2_ surface charge density vs. solution pH in the systems containing BY2 dye and surfactants with different ionic character.

The solid surface charge density changes as a function of the solution pH in the systems without and with examined dyes and those containing additionally surfactants with different ionic character were presented in Figure 8.

As can be seen in Figure 8, the point of zero charge (pzc) of the examined C/SiO_2_ composite without adsorbates is at pH about 3.1. Thus, practically, in the whole examined pH range (above pH 3.1), the solid surface is negatively charged. The adsorption of both dyes causes increase in the solid surface charge density and shift of pzc position towards the higher pH values (i.e., 6.5 for BB3 and 5.5 for BY2). The literature reports confirm that adsorption of small cations causes decrease in σ_0_ values, because the additional number of negatively charged surface groups are formed [45,46]. However, in the case of relatively big dye molecules, the positive charges in their structure are mostly located in the by-surface layer of the solution in the adsorption layer. This leads to the σ_0_ increase and such effect is more pronounced for BB3 molecules possessing higher molecular weight. Additionally, besides the electrostatic attraction between negatively charged composite surface and cationic dyes molecules, the hydrogen bonds can be formed between hydroxyl groups of the solid (–OH, –OH_2_^+^ and –O^-^) and nitrogen atoms in dye molecules [47,48]. The latter phenomenon leads to the exposure of the positive charges of the adsorbed dye molecules towards the bulk phase of the solution, which results in the observed increase in the surface charge density.

In the mixed systems of adsorbates (dye + surfactant), the noticeable changes in the course of σ_0_ dependencies occur. In the presence of anionic SDS, the further increase in the solid surface charge density (in comparison to the systems containing only dye) takes place (especially in the case of BY2 containing suspensions). In such a case the dye-SDS complexes are formed and they can be adsorbed on the solid surface. The negatively charged heads of anionic surfactant interact with positive charges of dye molecules, and thus such complexes undergo binding with the solid active sites mainly through hydrogen bonds (using for this purpose nitrogen atoms of dye molecules). In such a case the carbon tails of SDS surfactant are oriented towards the solution and the second adsorption layer of anionic surfactant can be formed with anionic heads directed into the solution.

The presence of cationic CTAB and nonionic Triton X-100 decreases significantly the dye adsorbed amounts. Such behavior confirm the competitive adsorption of these surfactants and dye molecules. The addition of nonionic surfactant has minimal effect on the σ_0_ values in the dye presence, whereas the addition of cationic surfactant resulted in noticeable decrease in the σ_0_ values in the pH range above pzc (i.e., 6.5 for BB3 and 5.5 for BY2). This proves the effective adsorption of CTAB molecules in the mixed surface layer, which leads to the formation of additional number of negatively charged surface groups [49]. There is the possibility of dye-Triton X-100 complexes formation through hydrophilic-hydrophobic interactions [50]; nevertheless, they remain mainly in the solution, and adsorption of a small number of them practically does not change the solid surface charge.

**Figure 9:**
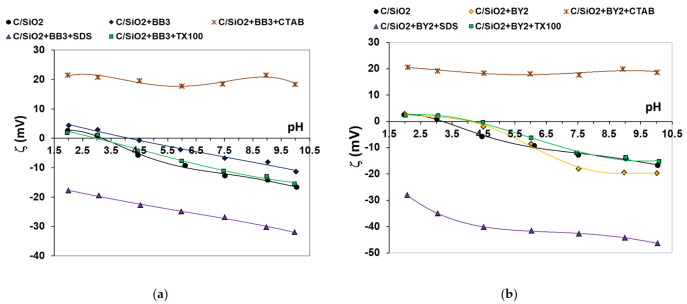
(**a**) Dependencies of the C/SiO_2_ zeta potential vs. solution pH in the systems containing BB3 dye and surfactants with different ionic character; (**b**) Dependencies of the C/SiO_2_ zeta potential vs. solution pH in the systems containing BY2 dye and surfactants with different ionic character.

The presence of adsorption layers of single and mixed adsorbates on the carbon-silica composite surface influences also the electrokinetic potential. The zeta potential changes as a function of the solution pH in the systems without and with examined dyes, and those containing additionally surfactants with different ionic character were presented in Figure 9. The isoelectric point (iep) for the C/SiO_2_ without additives occurs at pH about 3.3, which is in good agreement with the pzc value. The presence of both dyes (the small shift of iep to value of about 4.5 is observed) and dye+Triton X-100 mixed adsorbates changes inconsiderably the zeta potential of the solid particles. The changes in the zeta potential in the examined systems are the resultant of three phenomena accompanying the adsorption process: ionic character of adsorbate, shift of slipping plane from the solid surface and replacement of supporting electrolyte ions from the surface towards the diffuse part of electrical double layer [51].

In the case of anionic surfactant presence in the dye adsorption layer, the considerable shift of the slipping plane due to the dye-SDS complexes binding and formation of a second layer of surfactant molecules with anionic heads located in the slipping plane area are the more probable mechanisms of considerable reduction of zeta potential. On the other hand, the pronounced increase in electrokinetic potential in the mixed dye + CTAB adsorbates presence results from preferential adsorption of smaller cationic surfactant molecules (through the positive heads) and creation of CTAB adsorption multilayers with positive charges located in the slipping plane area [52].

## 4. Conclusions

The carbon-silica composite was applied for the removal of hazardous basic dyes such as C.I. Basic Blue 3 and C.I. Basic Yellow 2 from aqueous solutions. The batch equilibrium studies revealed high adsorption capacity of C/SiO_2_ towards dyes at the level of 924.6–1295.9 mg/g for BB3 and 716.3–733.2 mg/g for BY2 depending on temperature. There was observed increase in the adsorption capacity with increasing temperature in the BB3-C/SiO_2_ system. The Freundlich isotherm model describes equilibrium data better than Langmuir or Temkin ones. The physical nature of adsorption process was confirmed by the negative values of free energy and positive values of enthalpy. Additives such as anionic, cationic and non-ionic surfactants in the amount of 0.25 g/L reduced basic dyes adsorption on the composite surface. The adsorption of both dyes causes considerable increase in the solid surface charge density and noticeable increase in the zeta potential of C/SiO_2_ particles. The changes in electrokinetic parameters in mixed dye + surfactant systems of adsorbates proved the physical nature of their adsorption on the composite surface, mainly through hydrogen bonds. The specific structure of adsorption layers results on slipping plane shift and changes in ionic composition of surface and diffuse parts of electrical double layer formed at the solid/liquid interface.

Considering the above, the C/SiO_2_ composite can be considered as an effective adsorbent of impurities, including basic dyes from textile effluents or dyeing baths containing surfactants.

## Figures and Tables

**Table 1:** Isotherm models used for description of BY2—C/SiO_2_ and BB3—C/SiO_2_ systems.

Isotherm	Equation No.	Non-Linear Forms	Linear Forms
Langmuir	(3)–(4)	$q_{e}=\frac{k_{L}Q_{0}C_{e}}{1\;+\;C_{e}k_{L}}$	$\frac{C_{e}}{q_{e}}=\frac{1}{Q_{0}k_{L}}+\frac{C_{e}}{Q_{0}}$
Freundlich	(5)–(6)	$q_{e}=k_{F}C_{e}^{1/n}$	$l𝑜g\;q_{e}=l𝑜g\;k_{F}+\frac{1}{n}l𝑜g\;C_{e}$
Temkin	(7)–(8)	$q_{e}=\frac{RT}{b_{T}}lnAC_{e}$	$q_{e}=\;\left(\frac{RT}{b_{T}}\right)lnA+\left(\frac{RT}{b_{T}}\right)lnC_{e}$

where: *q*_e_—the amount of basic dye adsorbed per unit mass of C/SiO_2_ at equilibrium (mg/g), *C*_e_—the equilibrium concentration of solution (mg/L), *Q*_0_—the monolayer adsorption capacity (mg/g), *k_L_*—the Langmuir constant (related to the free energy of adsorption) (L/mg), *k*_F_ (mg^1−1/*n*^ L^1/*n*^/g) and 1/*n*—the Freundlich constants connected with adsorption capacity of adsorbent and the surface heterogeneity, respectively, R—the gas constant (8.314 J/mol K), T—the temperature (K), A (L/g) and b_T_ (J/mol)—the Temkin constants.

**Table 2:** Parameters of the Freundlich, Langmuir, and Temkin isotherm models calculated for the adsorption systems at 20–60 °C using linear regression.

Model	Parameters	BY2			BB3		
		Temperature (°C)					
		20	40	60	20	40	60
Langmuir	*Q*_0_ (mg/g) *k_L_* (L/mg) *R*^2^	1038.8 0.007 0.836	905.7 0.010 0.904	878.5 0.012 0.876	1252.3 0.026 0.904	1308.9 0.035 0.901	1443.2 0.053 0.914
Freundlich	$k_{F}$(mg^1−1/*n*^ L^1/*n*^/g) 1/*n* *R*^2^	29.32 0.558 0.977	34.50 0.531 0.991	58.11 0.434 0.970	81.56 0.531 0.984	144.25 0.419 0.948	243.99 0.338 0.946
Temkin	*b_T_* (J g/mol mg) *A* (L/mg) *R*^2^	14.77 0.137 0.838	15.23 0.160 0.894	18.64 0.362 0.812	12.38 0.608 0.847	14.86 1.952 0.760	15.22 6.565 0.773

**Table 3:** Parameters of the Freundlich, Langmuir, and Temkin isotherm models calculated for the adsorption systems at 20–60 °C using non-linear regression.

Model	Parameters	BY2			BB3		
		Temperature (°C)					
		20	40	60	20	40	60
Langmuir	*Q*_0_ (mg/g) *k_L_* (L/mg) *MPSD* *R*^2^ $R_{adj}^{2}$	891.8 0.008 0.405 0.946 0.930	680.8 0.017 0.255 0.897 0.867	686.3 0.019 0.699 0.874 0.838	1134.1 0.030 0.504 0.964 0.956	1251.7 0.035 0.949 0.962 0.954	613.5 1.75 2.350 0.471 0.383
Freundlich	$k_{F}$(mg^1−1/*n*^ L^1/*n*^/g) 1/*n* *MPSD* *R*^2^ $R_{adj}^{2}$	27.37 0.570 0.0879 0.991 0.988	34.09 0.533 0.032 0.993 0.991	55.41 0.442 0.109 0.978 0.971	77.70 0.543 0.083 0.994 0.992	124.55 0.456 0.287 0.983 0.979	223.25 0.357 0.365 0.970 0.965
Temkin	*b_T_* (J g/mol mg) *A* (L/mg) *MPSD* *R*^2^ $R_{adj}^{2}$	8.51 0.038 0.936 0.977 0.971	10.59 0.058 0.988 0.961 0.950	10.42 0.062 1.004 0.958 0.946	7.57 0.163 0.999 0.982 0.977	7.95 0.249 1.059 0.970 0.964	10.52 0.891 1.352 0.915 0.901

**Table 4:** Comparison of adsorption capacity of different adsorbents towards BB3 and BY2.

Kind of Adsorbent	*q_e_* (mg/g)	Ref.
	BB3	
Durian peel	49.5	[24]
Chitosan-based adsorbent	166.5	[25]
Sulfuric acid-activated montmorillonite	277	[26]
Fe_3_O_4_	7.474	-
PANI (polyaniline)	47.977	[17]
PANI/Fe_3_O_4_ composite	78.13	-
C/SiO_2_ composite	925–1296	This study
	**BY2**	
Natural untreated clay (Pasinler clay, originating from the eastern of Turkey)	833.3	[27]
Manure ash	1428.6	[28]
Granular activated carbon	598.8	[29]
Bagasse fly ash Mesoporous carbon oxidized at 30–60 °C C/SiO_2_ composite	31.17 172–355.2 716–733	[30] [31] This study

**Table 5:** Thermodynamic parameters of BB3 and BY2 adsorption on C/SiO_2_ composite.

T (°C)	K_c_ (L/g)	ΔH^𝑜^ (kJ/mol)	ΔS^𝑜^ (kJ/mol)	ΔG^𝑜^ (kJ/mol)
	BB3			
20	9.7	12.6	61.6	−22.4
40	13.0			−24.7
60	17.9			−27.1
	**BY2**			
20	2.6	1.6	13.3	−19.2
40	2.5			−20.4
60	2.7			−21.9

## Data Availability

The data presented in this study are available in this article.
